# MRI versus relapse: optimal activity monitoring for management of progressive multiple sclerosis

**DOI:** 10.1093/braincomms/fcaf010

**Published:** 2025-01-20

**Authors:** Gavin Giovannoni, Suzannah Hetherington, Eddie Jones, Patricia Dominguez Castro, Himanshu Karu, Soudeh Ansari, Goeril Karlsson, Virginia de las Heras, Carol Lines

**Affiliations:** The Faculty of Medicine and Dentistry, Blizard Institute, Queen Mary University of London, London E1 2AT, UK; Novartis Pharma AG, Basel CH-4056, Switzerland; Adelphi Real World, Manchester SK10 5JB, UK; Novartis Ireland Ltd, Dublin D04A9N6, Ireland; Novartis Healthcare Pvt. Ltd, Hyderabad 500081, India; Novartis Pharmaceuticals, Cambridge, MA 02139, USA; Novartis Pharma AG, Basel CH-4056, Switzerland; Novartis Pharma AG, Basel CH-4056, Switzerland; Novartis Pharma AG, Basel CH-4056, Switzerland

**Keywords:** MRI activity, relapses, inflammatory disease markers, active secondary progressive multiple sclerosis, non-active secondary progressive multiple sclerosis

## Abstract

Secondary progressive multiple sclerosis is often categorized as ‘active’/‘non-active’ based on inflammatory activity on MRI, or relapse; however, the value of MRI/relapse as indicators of disease activity in real-world and clinical trial settings merits further investigation. We separately analysed retrospective data from patients with clinically diagnosed secondary progressive multiple sclerosis in the Adelphi Real-World Disease Specific Programme (a cross-sectional survey) in multiple sclerosis (Adelphi: *n* = 2554) and the placebo group of the Phase III EXploring the efficacy and safety of siponimod in PAtients with secoNDary progressive multiple sclerosis (EXPAND) trial, [EXPAND-PBO (placebo group of the EXPAND): *n* = 546] to assess: differences between active/non-active disease in the real-world (characteristics; monitoring); the value of MRI and relapse to indicate disease activity; and the number and characteristics of non-active patients with disease activity in the clinical study. In Adelphi, 1889 patients had ‘active’ disease (≥1 relapse in the year before index date and/or ≥1 new lesion on most recent MRI) versus 665 with ‘non-active’ disease (no relapses in the previous year and no new lesions on MRI); median age was 48 versus 53 years; 73.5 versus 87.8% had moderate-to-severe disease; 75.7 versus 54.3% were taking disease-modifying treatment; 87.7 versus 58.7% had received an MRI in the past year. Most active cases (*n* = 1116; 59.1%) were identified by MRI versus 239 (12.7%) by relapse and 534 (28.3%) by MRI plus relapse. In EXPAND-PBO, 263 patients were classified ‘active’ (≥1 relapse in 2 years before screening and/or ≥1 gadolinium-enhancing lesion) and 270 ‘non-active’ (no relapse in the 2 years before screening and no gadolinium-enhancing lesion[s]) at baseline; similar proportions of these groups had received disease-modifying treatment prior to placebo: 77.2 and 80.7%. Of non-active patients, 53.0% had disease activity on study; in these patients, 74.1% had disease activity identified by MRI, 8.4% by relapse, and 17.5% by MRI plus relapse. In patients classified non-active at baseline: age and percentage with Expanded Disability Status Scale score 6.0–6.5 were similar between patients with disease activity on study versus patients who remained non-active: 48 versus 52 years; 49.7 versus 56.7%, respectively. In real-world and clinical trial settings, MRI could be a better option than relapse for the identification of disease activity. However, in the real-world, fewer non-active patients had received an MRI in the last year than active patients, which is concerning given that most disease activity in EXPAND-PBO was identified via MRI. We highlight difficulties in consistently identifying disease activity and the negative implications of infrequent monitoring of non-active disease.

## Teaser

Giovannoni *et al.* sought to determine the relative contributions of MRI activity and/or relapses in defining secondary progressive multiple sclerosis (SPMS) disease activity in real-world and clinical trial settings. Their findings show that MRI appears more sensitive than relapses in measuring disease activity in patients with active SPMS.

## Graphical Abstract

**graphical abstract:**
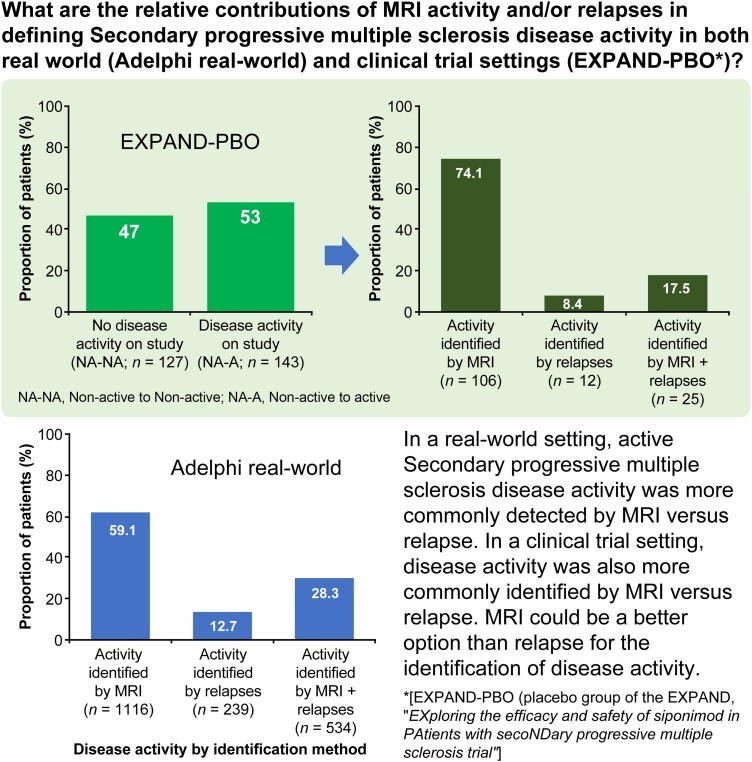

## Introduction

Multiple sclerosis (MS) is considered a disease continuum, with inflammatory features most prominent in the early stages of the disease, and degenerative processes increasing later in the disease course.^1,2^

Despite key differences between the relapsing-remitting and secondary progressive multiple sclerosis (SPMS) phenotypes, the similarities between the pathological features and disease mechanisms and the lack of a clear ‘transition point’ between these phenotypes, point to a gradual shift in the predominant mechanism of disability accrual, rather than a binary switch.^3^

More than 60% of patients with untreated relapsing-remitting multiple sclerosis (RRMS) gradually transition (20–40% within 10 years; up to 50% within 20 years) to SPMS; a phenotype that has a poor prognosis, typically fewer treatment options and is associated with cognitive decline.^4-6^ Older age at disease onset is a key prognostic factor for the development of secondary progressive disease^5^ and accrual of disability.^7^

Although it is widely recognized that relapse leads to disability and, early in the disease course, relapse-associated worsening (RAW) is a significant driver of disability, disease worsening is also driven by the insidious progression that occurs independently of relapse activity (PIRA).^2,7,8^ Growing evidence demonstrates that PIRA is responsible for up to half of disability accumulation in adults with RRMS, and as the disease evolves into SPMS, PIRA becomes the dominant driver of disability.^7-9^ Importantly, SPMS is evident when disability has accumulated following the exhaustion of functional neurological reserves.^10^

Most sustained and non-sustained PIRA in older patients with prolonged disease and greater disability are associated with transition to secondary progression (SP), potentially indicating the onset of the progressive phase clinically.^11^ SPMS is clinically identified retrospectively, based on the review of the patient's disease history and identification (after an initial relapsing-remitting course) of disability accrual independent of relapses (although RAW may still occur).^4,12,13^ Certain imaging observations may be useful for retrospective diagnosis of secondary progressive disease (i.e. detection of active spinal cord lesions on follow-up MRI scans)^14^; however, no current diagnostic imaging marker can predict the evolution of relapsing-remitting to progressive disease.^15^ People living with SPMS are often further categorized as having an ‘active’ or ‘non-active’ phenotype based on the presence/absence of inflammatory disease activity revealed by clinical relapses and/or new lesions detected via MRI.^12^ While there are disease-modifying treatments (DMTs) for patients with active disease those without active disease typically do not receive a DMT; moreover, only a few countries have approved therapies for the treatment of SPMS.^16,17^

It is recommended that disease activity status should be evaluated annually, at minimum, by assessing whether a relapse has occurred, or whether the presence of disease activity on MRI has been detected in the past 12 months; however, MRI monitoring has yet to be established or standardized for patients with PIRA.^12,18^ The sensitivity of MRI versus relapse to reveal inflammation related to disease progression, and the extent to which these measures are redundant with each other has a practical application in how patients should be monitored.^19^ Thus, it is important to determine whether the most sensitive and appropriate methods for identification of disease activity are being implemented in real-world clinical practice and whether there are any differences in the monitoring of patients thought to have active or non-active disease.

To better understand the differences in disease activity between patients with ‘active’ or ‘non-active’ SPMS, the role of MRI and relapses in measuring disease activity, and the characteristics of patients with SPMS associated with disease activity, we separately analysed data from the Adelphi Real-World Disease Specific Programme (DSP) in MS (referred to hereafter as ‘Adelphi’) and the placebo group of the EXploring the efficacy and safety of siponimod in PAtients with secoNDary progressive multiple sclerosis (EXPAND) (NCT01665144) Phase 3 trial of siponimod in SPMS [referred to hereafter as placebo group of the EXPAND (‘EXPAND-PBO’)].

Real-world differences in patients with active and non-active SPMS were evaluated using real-world data from Adelphi. The relative contributions of MRI and occurrence of relapse in defining disease activity were investigated using real-world data from Adelphi and clinical trial data from EXPAND-PBO. Lastly, to better understand potential predictors of active disease and optimal monitoring of at-risk patients, the characteristics and monitoring of a subset of patients in the placebo group of the EXPAND trial with non-active SPMS at baseline who subsequently developed active disease on the study were evaluated.

## Materials and methods

### Datasets and inclusion criteria

#### Adelphi real-world DSP in MS

Data were retrieved from The Adelphi DSP database (described previously). In brief, the database was constructed using information from patient record forms (PRFs) completed by physicians and patient/caregiver questionnaires (self-completed by each patient or their caregiver).^20^

The Adelphi DSP in MS was a cross-sectional, non-interventional, multinational (Europe and United States) survey of neurologists and their patients with MS. Clinical data were retrospectively collected in yearly waves (from 1 January 2011 to 31 December 2019) from ∼700 neurologists, each asked to provide data from their next 10 consultations with patients diagnosed with MS. Criteria for inclusion in the Adelphi dataset in the current analysis were age ≥ 18 years old at index date, clinical diagnosis of SPMS and classified as having either active or non-active disease. Patients with missing data that prevented classification in terms of disease activity were excluded.

#### Placebo group of the expand trial: EXPAND-PBO

Data were collected during the EXPAND trial (5 February 2013 to 2 June 2015), conducted at 292 hospital clinics and specialized MS centres in 31 countries.^21^

Full methods and results for EXPAND have been published previously.^21^ In brief, EXPAND was an event-driven and exposure-driven, double-blind, Phase III trial of the efficacy and safety of siponimod versus placebo. Patients aged 18–60 years with SPMS and an expanded disability status scale (EDSS) score of 3.0–6.5 were eligible for enrolment.^21^ Due to the event-driven design, the duration of study in EXPAND varied between patients (median: 21 months; range: <1–37 months).^21^ The current analyses are based on data from the placebo group in EXPAND only. Patients with missing data that prevented classification in terms of disease activity were excluded.

## Patient consent

The Adelphi Real-World DSP in MS was a real-world multinational survey of neurologists and their patients with MS and as such did not require any formal ethical approval [the study protocol (reference number AG AG8651) was submitted to the Western Institutional Review Board, that determined ethical approval was not required and an ethics waiver was issued accordingly]. As previously described, in all study countries, DSP fieldwork teams adhered to national data collection regulations.^20^ The Adelphi Real-World DSP in MS was conducted in accordance with the European Pharmaceutical Market Research Association (EphMRA) Code of Conduct. All data were collected following procedures with Ethics Committee approval and data were fully de-identified prior to receipt by Adelphi Real-World. The respondents provided informed consent for the use of their anonymized and aggregated data for research and publication in scientific journals. All data, methodology, materials, and data analysis that support the findings of this survey are the intellectual property of Adelphi Real-World, and as such, no administrative permissions were required to access or use the data. For EXPAND-PBO, the EXPAND trial adhered to the International Conference on Harmonization Guidelines for Good Clinical Practice and to the Declaration of Helsinki and Institutional Review Boards or Ethics Committees approved the protocol at all sites; all patients included in the analysis gave written informed consent before commencing the study.^21^

### Study design

#### Outcome measures

*Post hoc* analyses were conducted to investigate demographics and clinical characteristics by disease activity status (active versus non-active) at index date (Adelphi) or baseline (EXPAND-PBO), and the demographics and clinical characteristics of patients with and without on-study disease activity in a subset of patients with non-active disease at baseline in EXPAND-PBO. Index date is defined as the date of PRF submission.

In Adelphi, data collated from PRFs included patient age, sex, duration of disease, EDSS score (as assessed by clinicians^22^), clinician-assessed disease severity (unknown, very mild, mild, moderate or severe), use of DMTs, and the number of MRI scans received during the 12 months prior to the index date (date of PRF survey submission). Very mild disease implies no impact on physical and occupational functioning. With mild disease, study subjects have neurological impairments with no effect on physical function. Subjects with moderate disease have neurological disability with an impact on physical function, but study subjects are independent. Subjects with severe disease have physical neurological disability of sufficient severity to impact independent function.

For EXPAND-PBO, baseline data included age, sex, duration of disease, EDSS score (minimum, 0; maximum, 10, as assessed by the treating clinician^22^), MS severity score (MSSS; minimum, 0.01; maximum, 9.99),^23^ and the number of relapses during the 12 months prior to the study start date. MRI scans were conducted at baseline, after 12, 24, and 36 months, and at the end of treatment, and the occurrence of relapses (assessed at/outside of clinic visits) was recorded.^21^

#### Categorical classification of active/non-active SPMS

##### Adelphi real-world DSP in MS

Patients with evidence or no evidence of inflammatory disease activity were categorized as active or non-active, respectively. Inflammatory disease activity was defined as the presence of ≥1 gadolinium-enhancing (Gd+) or new T1 or T2 lesion in the most recent MRI scan, and/or the occurrence of ≥1 relapse (as reported in the PRF by the participating clinician) during the 12 months prior to the index date.

##### EXPAND-PBO

Patients with evidence or no evidence of inflammatory disease activity were categorized *post hoc* as having active or non-active disease, respectively, according to data collected at baseline in EXPAND. Inflammatory disease activity was defined as the presence of ≥1 Gd+ T1 lesion in the baseline MRI scan, and/or the occurrence of ≥1 relapse during the 2 years prior to screening. The dates of any relapses within the 2 years prior to screening were reported in the study case report form. At baseline, if a patient had a missing record for relapse or Gd+ T1 lesion in the previous 2 years or was missing a Gd+ assessment on the MRI baseline scan, the status of the patient was marked as ‘missing’ and not included in the analysis. Inflammatory disease activity on the study was identified during follow-up through lesion activity detected on MRI scans (Gd+ T1 and/or new/enlarging T2 lesion), or the occurrence of relapse, or both.

### Statistical analyses

Patient demographics and clinical characteristics are presented using descriptive statistics. For descriptive analyses, continuous variables were summarized using standard summary statistics [mean, SD, range (*n*), and inter-quartile range (25%, 75%)]. Categorical variables were summarized using frequency counts and percentages. No statistical comparisons between the Adelphi and EXPAND-PBO datasets or between groups in each dataset have been made.

## Results

### Baseline demographics

#### Adelphi real-world DSP in MS

Among 3580 patients diagnosed with SPMS between 2011 and 2019, 1889 patients were identified as having active disease and 665 patients as having non-active disease. At the index date (1 January 2011), the median age of the active disease group was lower than the non-active disease group (48.0 and 53.0 years) (Table 1). Approximately two-thirds of patients were female in both the active (64.5%) and non-active groups (62.9%) and the median age at initial diagnosis of MS was similar between groups (37.4 and 37.0 years); however, the active group tended to have a shorter disease duration (median time since diagnosis: 9.1 versus 13.2 years) (Table 1). The median age of diagnosis of SPMS was slightly lower in the active disease group versus the non-active disease group (43.4 versus 46.0 years), and the median time since diagnosis of secondary progressive disease was also slightly lower in this group (3.0 versus 5.1 years) (Table 1).

**Table 1:** Demographics at index date in the Adelphi DSP and at baseline in EXPAND-PBO

	Adelphi DSP (*n* = 2554)^a^		EXPAND-PBO (*n* = 533)^a^	
	Active (*n* = 1889)	Non-active (*n* = 665)	Active (*n* = 263)	Non-active (*n* = 270)
Median age, years (range)^b^	48.0 (18.0–86.0)	53.0 (21.0–84.0)	48.0 (21.0–60.0)	50.0 (29.0–61.0)
18–40 years, *n* (%)	445 (23.6)	68 (10.2)	61 (23.2)	39 (14.4)
>40 years, *n* (%)	1444 (76.4)	597 (89.8)	202 (76.8)	231 (85.6)
Sex, *n* (%)^b^				
Female	1219 (64.5)	418 (62.9)	166 (63.1)	151 (55.9)
Male	668 (35.4)	247 (37.1)	97 (36.9)	119 (44.1)
Multiple sclerosis diagnosis				
Median age at diagnosis, years (range)	37.4 (8.7–77.0)	37.0 (13.9–77.5)		
Median time since diagnosis, years (range)	9.1 (0–45.0)	13.2 (0–46.0)	10.3 (0.4–33.2)	12.5 (0.4–39.4)
Secondary progressive multiple sclerosis diagnosis				
Median age at diagnosis, years (range)	43.4 (8.7–80.2)	46.0 (18.1–77.5)		
Median time since diagnosis, years (range)	3.0 (0–39.0)	5.1 (0–39.0)	1.9 (0.1–21.7)	3.0 (0.3–18.3)

DSP, disease specific programme.

^a^Adelphi: only patients with active/non-active classification were included; EXPAND-PBO: if a patient had a missing value for either super imposed relapse or Gd+ T1 lesion at baseline, the status for this patient was marked as ‘missing’ and was not included in the analysis.

^b^Total may not sum due to rounding.

#### EXPAND-PBO

Of the 546 patients enrolled in the EXPAND trial placebo arm, a total of 263 and 270 were categorized as having active and non-active disease at baseline, respectively, and were included in the analysis; 13 patients enrolled did not meet the inclusion criteria and were not included in the study. The median age of the two groups was similar (48.0 and 50.0 years), and 76.8 and 85.6% of patients in each group were aged over 40 years, respectively (Table 1). There was a slightly higher proportion of females than males in both the active (63.1%) and non-active (55.9%) groups. The median time since the initial diagnosis of MS was slightly shorter for patients with active disease versus non-active disease: 10.3 versus 12.5 years, as was the median time since SPMS diagnosis: 1.9 versus 3.0 years, respectively (Table 1).

### Clinical characteristics and MRI utilization in patients with active and non-active disease

#### Adelphi real-world DSP in MS

**Figure 1:**
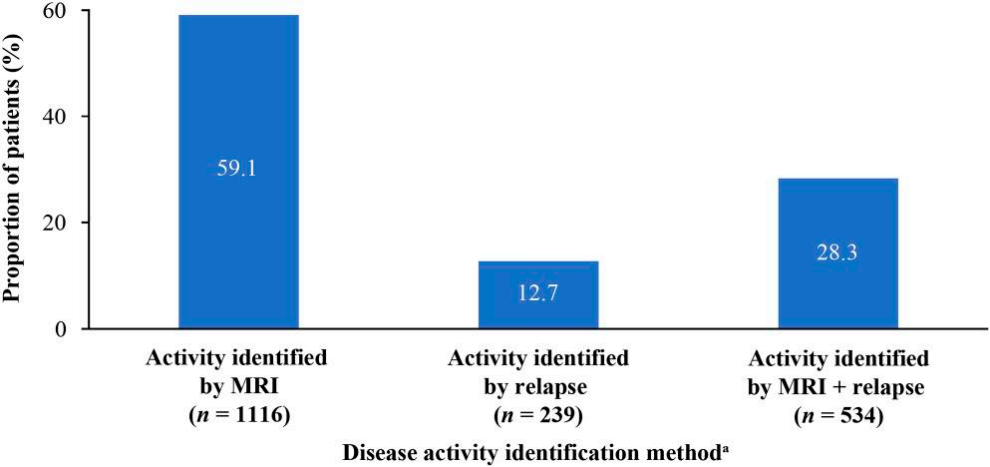
**Identification of disease activity in 1889 patients with active secondary progressive MS at index date (Adelphi real-world DSP in MS).** ^a^A total of 496 patients were missing relapse data at baseline; 232 patients had no MRI in last 12 months. Disease activity was defined as the presence of ≥1 Gd+ or new T1 or T2 lesion in the most recent MRI scan, and/or the occurrence of ≥1 relapse (as reported in the PRF by the participating clinician) during the 12 months prior to the index date.

Mean change in EDSS scores in the past 12 months in the active and non-active disease groups were 0.4 (SD 0.6) and 0.2 (0.5), respectively. Similar proportions of each group were classified as having moderate disease according to physician assessment (53.2 and 57.3%, respectively), whereas the proportion with severe disease was ∼10% higher in the non-active group (20.3 and 30.5%, respectively, Table 2). At the index date, approximately three-quarters (75.7%) of patients with active disease were taking a DMT, compared with approximately half (54.3%) of those with non-active disease. During the 12 months prior to the index date, 93.2% of patients had a neurological examination (95.5 and 93.2% of the active and non-active disease groups, respectively); and almost three-quarters of patients (72.4%) had received an MRI scan; however, a greater proportion of the active disease group had received an MRI than the non-active group (87.7 versus 58.7%, respectively, Table 2). During the same time period, 32.8% of patients with active disease had not had a relapse, approximately a quarter (25.8%) of patients had one relapse, 15.1% had ≥2 relapses, and approximately a quarter (26.3%) were missing relapse data (Table 2). By definition, no patient in the non-active group had a relapse during the 12 months prior to the index date. Among the 1889 patients classified with active disease, 1116 cases (59.1%) were identified on the basis of inflammatory activity on the most recent MRI, 239 (12.6%) by the occurrence of relapse during the 12 months prior to the index date, and 534 (28.3%) by both MRI and relapse criteria (Fig. 1).

**Table 2:** Clinical characteristics and MRI utilization according to disease status at index date in the Adelphi DSP and at baseline in EXPAND-PBO

	Adelphi DSP (*n* = 2554)^a^		EXPAND-PBO (*n* = 533)^b^	
	Active (*n* = 1889)	Non-active (*n* = 665)	Active (*n* = 263)	Non-active (*n* = 270)
EDSS score, mean (SD)	*n* = 1657	*n* = 624		
	4.8 (1.8)	5.4 (1.8)	5.4 (1.0)	5.4 (1.0)
Change in EDSS score in the past 12 months, mean (SD)	*n* = 1462	*n* = 605		
	0.4 (0.6)	0.2 (0.5)		
Patients with moderate or severe disease based on physician's assessment, *n* (%)	1389 (73.5)	584 (87.8)		
Moderate	1005 (53.2)	381 (57.3)		
Severe	384 (20.3)	203 (30.5)		
Patients who had an MRI scan in the past 12 months, *n* (%)	1657 (87.7)	390 (58.7)		
Number of MRI scans conducted				
In the past 12 months	1848	576		
Per person in the past 12 months, mean (SD)	1.24 (0.8)	0.9 (0.8)		
Median number of relapses in the past 12 months, *n* (range)	*n* = 1393	*n* = 665		
	1.0 (0–8.0)	N/A	0 (0–4.0)	N/A
Number of relapses in the past 12 months, *n* (%)				
0	620 (32.8)	665 (100)	134 (51.0)	270 (100)
1	488 (25.8)	N/A	111 (42.2)	N/A
2–3	253 (13.4)	N/A	16 (6.1)	N/A
≥4	32 (1.7)	N/A	2 (0.8)	N/A
Missing	496 (26.3)	N/A	0	N/A
Median number of Gd+ T1 lesions, *n* (range)			258 (0–65.0)	0 (0–0)
Patients taking DMT at index date, *n* (%)	1430 (75.7)	361 (54.3)		
Patients taking DMT prior to receipt of randomized treatment, *n* (%)			203 (77.2)	218 (80.7)
Time between last DMT and first dose of study drug (patients taking DMT prior to receipt of randomized treatment only), *n* (%)^c^				
≤3 months			65 (24.7)	77 (28.5)
>3–≤6 months			23 (8.7)	29 (10.7)
>6 months–≤1 year			37 (14.1)	26 (9.6)
>1–≤2 years			16 (6.1)	23 (8.5)
>2 years			62 (23.6)	63 (23.3)

DMT, disease-modifying treatment; DSP, disease specific programme; EDSS, expanded disability status scale; Gd+, gadolinium-enhancing; N/A, not applicable; SD, standard deviation.

^a^Not all patient data are available for all categories; where the total populations are not *n* = 1889 or *n* = 665 for active and non-active groups, patient totals for ‘non-missing’ data are listed.

^b^If a patient had a missing value for either superimposed relapse or Gd+ T1 lesion at baseline, the status for this patient was marked as ‘missing’ and was not included in the analysis.

^c^If the end date for DMT was missing, patients were considered to have stopped DMT on the day prior to the first dose of randomized treatment; similarly, if DMT were discontinued after the first dose of randomized treatment, patients were treated as if they had been stopped 1 day before the first dose of randomized treatment.

#### EXPAND-PBO

At baseline, the mean (SD) EDSS scores in the active and non-active groups were the same: 5.4 (1.0) (Table 2). In the 12 months prior to screening, approximately half (51.0%) of the patients with active disease had no relapses, 42.2% had 1 relapse and 6.9% had ≥2 relapses (Table 2); in the 24 months prior to screening, 23.2% of patients had no relapses, 39.5% had 1 relapse and 37.3% had >1 relapse. By definition, no patient with non-active disease had a relapse in the 24 months prior to screening, or any Gd+ T1 lesions at baseline. At baseline, 77.2% of patients with active disease and 80.7% of patients with non-active disease had received ≥1 DMT prior to receipt of randomized treatment (Table 2); a categorical analysis of the length of time between the last DMT and the first dose of randomized treatment (i.e. placebo) did not indicate any meaningful differences between the active and non-active groups for this variable at baseline (Table 2).

**Figure 2:**
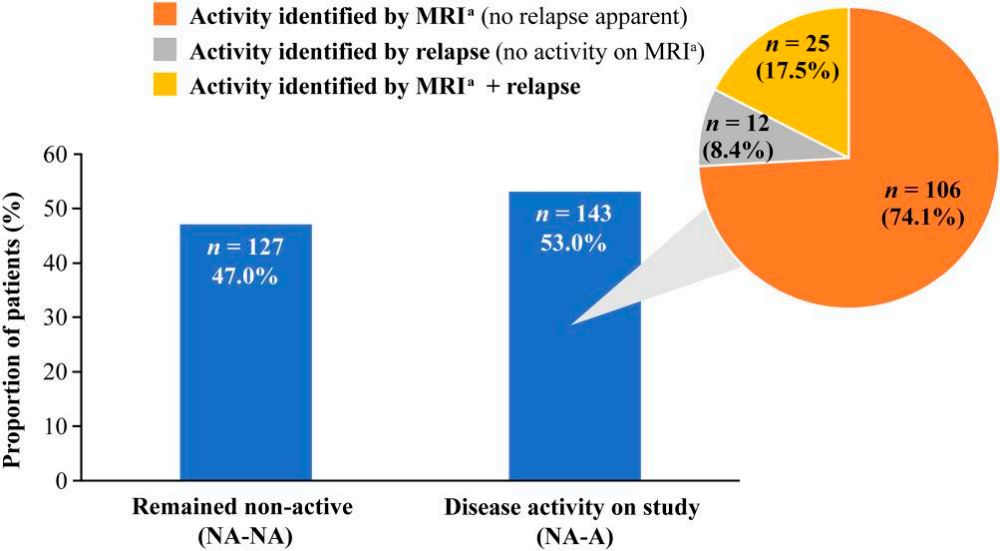
**Patients with SPMS with and without disease activity on study in the EXPAND-PBO group plus method of disease activity identification.** Bar chart shows percentage of disease activity in the study in 270 patients classified with ‘non-active’ disease at baseline; pie chart shows the method of disease activity detection in the 143 patients who had disease activity in study. ^a^Definition of disease activity in the study included evidence of Gd+ T1 and/or new/enlarging T2 lesions on MRI. NA–NA, non-active–non-active (i.e. patients categorized non-active at baseline and remained non-active on the study); NA–A, non-active–active (i.e. patients categorized non-active at baseline with disease activity on study).

Among the 270 patients categorized as non-active at baseline, disease activity was identified during study follow-up in 143 patients (53.0%) [herein referred to as the non-active to active (NA–A) group]. Of the patients in the NA–A group, the majority (74.1%) had disease activity identified by inflammatory activity on MRI with no relapse apparent, 8.4% had disease activity identified by relapse with no inflammatory activity on MRI, and the remaining 17.5% had disease activity identified by both MRI and relapse criteria (Fig. 2). A total of 127 patients remained non-active in the study [herein referred to as the non-active–non-active (NA–NA) group] (Fig. 2).

Patients in the NA–A group had a slightly lower median age at baseline than patients in the NA–NA group (median 48.0 and 52.0 years), with 79.7% of the NA–A group aged over 40 years old versus 92.1% of the NA–NA group (Table 3). The median disease duration since diagnosis of MS in each of these groups was 11.5 and 12.9 years, respectively, and the median time since diagnosis of SPMS was also shorter in the NA–A group (2.7 and 3.8 years) (Table 3).

**Table 3:** Patient baseline data by presence/absence of on-study disease activity in EXPAND-PBO

	Non-active at baseline (*n* = 270)^a^		
On study follow-up	Disease activity identified (‘NA–A’)	Remained non-active (‘NA–NA’)	*P-*value
	(*n* = 143)	(*n* = 127)	
Median age, years (range)	48 (29.0–61.0)	52 (30.0–61.0)	
18–40 years, *n* (%)	29 (20.3)	10 (7.9)	
>40 years, *n* (%)	114 (79.7)	117 (92.1)	
*P-*value (CHI)			*0.0038*
Sex, *n* (%)			
Female	81 (56.6)	70 (55.1)	
Male	62 (43.4)	57 (44.9)	
*P-*value (CHI)			*0.8011*
Median disease duration, years (range)	11.5 (0.4–39.4)	12.9 (0.5–39.2)	
*P-*value (WR)			*0.1084*
Time since conversion to secondary progressive multiple sclerosis, years (range)	2.7 (0.5–18.2)	3.8 (0.3–18.3)	
*P-*value (WR)			*0.0711*
EDSS score, mean (SD)	5.3 (1.0)	5.5 (1.0)	
*P-*value (WR)			*0.2939*
EDSS categories, *n* (%)			
<3.0	0	0	
3.0–3.5	14 (9.8)	11 (8.7)	
4.0–4.5	30 (21.0)	24 (18.9)	
5.0–5.5	27 (18.9)	20 (15.7)	
6.0–6.5	71 (49.7)	72 (56.7)	
>6.5	1 (0.7)	0	
*P-*value (MHT)			*0.4341*
MSSS, mean (SD)	6.0 (1.7)	5.7 (1.8)	
*P-*value (WR)			*0.2275*
Patients taking DMT prior to receipt of randomized treatment, *n* (%)	116 (81.1)	102 (80.3)	
*P*-value (CHI)			*0.8672*
Time between last DMT and first dose of study drug (patients taking DMT prior to receipt of randomized treatment only), *n* (%)^b^			
≤3 months	36 (25.2)	41 (32.3)	
>3–≤6 months	19 (13.3)	10 (7.9)	
>6 months–≤1 year	14 (9.8)	12 (9.4)	
>1–≤2 years	13 (9.1)	10 (7.9)	
>2 years	34 (23.8)	29 (22.8)	
*P*-value (MHT)			*0.6718*
Median time since onset of most recent relapse, months (range)	54.1 (18.3–315.9)	67.4 (20.9–288.3)	
*P*-value (WR)			*0.0862*

CHI, χ^2^ test; DMT, disease-modifying treatment; DSP, disease specific programme; EDSS, expanded disability status scale; MSSS, multiple sclerosis severity score; NA–NA, non-active–non-active (i.e. patients categorized non-active at bassline and remained non-active on study); NA–A, non-active–active (i.e. patients categorized non-active at bassline with subsequent disease activity on study); MHT, Mantel Haenszel test for non-zero correlation; WR, Wilcoxon rank sum test. *P* value represented in italics.

^a^If a patient had a missing value for either superimposed relapse or Gd + T1 lesion at baseline, the value that was not missing was recorded and the patient was included in the analysis.

^b^If the end date for DMT was missing, patients were considered to have stopped DMT on the day prior to first dose of randomized treatment; similarly, if DMT were discontinued after first dose of randomized treatment, patients were treated as if they had been stopped 1 day before the first dose of randomized treatment.

At baseline, a slightly smaller proportion of the NA–A group (49.7%) than the NA–NA group (56.7%) had an EDSS score of 6.0–6.5. A similar proportion of patients in both the NA–A and NA–NA groups had received ≥1 DMT prior to randomized treatment (81.1 and 80.3%, respectively); and a categorical analysis of the length of time that DMT therapy was stopped prior to randomized treatment did not indicate any differences between the NA–A and NA–NA groups (Table 3). The mean (SD) MSSS at baseline was similar between the NA–A and NA–NA groups: 6.0 (1.7) and 5.7 (1.8).

Per definition, no patients in either the NA–A or NA–NA group had Gd+ T1 lesions in the baseline MRI scan, or relapse during the 2 years prior to screening. The median time since the onset of the most recent pre-study relapse was 54.1 months in the NA–A group compared with 67.4 months in the NA–NA group (Table 3).

## Discussion

In both Adelphi and EXPAND-PBO, patients with evidence or no evidence of inflammatory disease activity were categorized as active or non-active, respectively. In both the real-world and in clinical trial settings, baseline demographics and clinical characteristics were comparable between the active and non-active SPMS groups. In the Adelphi cross-sectional survey of real-world clinical practice, patients clinically diagnosed with active SPMS were younger overall than patients with non-active disease (difference in median values: 5 years), and 76.4 versus 89.8% of these patient groups were older than 40 years. The median age at initial diagnosis of MS was similar between patients with active and non-active disease (37.4 versus 37.0 years); however, patients with active disease were slightly younger at the time of their SPMS diagnosis (difference in median values: 2.6 years). In the EXPAND clinical trial placebo group, patients with active disease tended to be slightly younger overall than patients with non-active disease (difference in median values: 2 years), and 76.8 versus 85.5% were older than 40 years. Based on time since diagnosis of MS, patients with active disease had a somewhat shorter disease duration than patients with non-active disease (difference in median values: 2.2 years); however, the median time since diagnosis of secondary progressive disease was similar between the groups (difference in median values: 1.1 years). Although patients with SPMS were younger than patients with non-active disease in both real-world and clinical trial settings, the lack of other notable demographic or clinical differences (including age at diagnosis of MS) between the active and non-active groups highlights the limitations in our current understanding of differences that underlie active and non-active disease, and in determining predictors of active disease.

Despite a smaller proportion of the active than the non-active group having moderate–severe disease at the index date (74 versus 88%), approximately half the non-active group were prescribed DMT at the index, compared with approximately three-quarters of the active group. This may be reflective of a more general tendency for patients with non-active disease not to be prescribed a DMT, largely due to the lack of approved therapies for the treatment of SPMS.^3,16^

In both Adelphi and EXPAND-PBO, inflammatory activity on MRI was found to be a more sensitive tool to identify disease activity than relapse occurrence. In the real-world dataset, the majority of the group classified as having ‘active disease’ at index date had disease activity identified on the basis of inflammatory disease activity on MRI (59.1%), whereas 28.3% had disease activity identified by relapse and MRI criteria, and just 12.7% had disease activity identified by relapse alone (although it should be noted that 496 of the 1889 patients classified with active disease were lacking data on relapse). Further, in total, 87.7% of the active group versus 58.7% of the non-active group had received an MRI scan in the 12 months prior to index. Therefore, it appears that clinicians tended to use MRI to monitor inflammatory disease activity less frequently in patients who fulfilled the criteria for non-active disease at the index than in patients who fulfilled the criteria for active disease. Compared with patients in the active group, the non-active patients were older, and a smaller proportion of the group had been prescribed a DMT (active, 75.7%; non-active, 54.3%); thus, the lower frequency of MRI in non-active patients may reflect a systemic, cognitive bias among clinicians to monitor patients with these characteristics less frequently. Evidence for reduced MRI monitoring in patients with non-active SPMS in the real-world setting is a concern; such patients should be monitored closely, as this would increase the chances of detecting and treating new disease activity in this population.

Our findings highlight the importance of, and need for, serial monitoring of patients with non-active disease, to improve the detection of inflammatory disease activity and facilitate timely treatment to prevent further disability accumulation. Few treatments for non-active disease exist: a few countries have approved therapies for SPMS that can be used without considering activity status, and in other regions, no DMTs are approved for this population. However, our findings show that many patients categorized as non-active may develop subsequent disease activity and could potentially benefit from treatments currently restricted to patients with active SPMS. In the Adelphi Real-World DSP in MS (conducted in the European Union and USA), over half of the patients deemed non-active were not being treated with a DMT, while the EXPAND-PBO group analysis (with observation on the study of up to 37 months) raises the possibility that disease activity could be revealed in patients thought to be non-active if assessed more frequently with MRI. The high proportion of untreated patients in a real-world setting reflects the paucity of treatment options for SPMS. Although incomplete recovery from relapse is associated with disability accrual in patients with MS,^24^ it is apparent that later in the disease course PIRA becomes the main driver of disability accumulation^7^; therefore, there is a need for therapies that address mechanism(s) of progression that are independent of relapse activity, via new therapeutic targets; this may ultimately lead to better treatment for patients.

The growing body of research demonstrating that relapses are not the only cause of insidious worsening in progressive MS,^2,7-9^ combined with our findings suggesting that the occurrence of relapse is not a reliable indicator of disease activity, strongly support the hypothesis that MRI is a more sensitive measure. The EXPAND-PBO analysis revealed that, in the 270 patients deemed to have non-active disease at baseline, 53.0% had disease activity detected in the study; of which 74.1% of cases were identified by MRI activity without apparent relapse; this hypothesis is further supported by the Adelphi DSP Real-World data, where 59.1% of all patients classified as active at the index date had the disease activity identified on the basis of MRI activity alone.

It has been argued that, in order to accelerate treatment decisions and improve patient outcomes, definitions of disease activity should be refined to account for all parameters that consistently predict relapse and disability progression.^25^ There is also a need for tools to improve the identification of SPMS in clinical practice.^3^ To different extents, both RAW and PIRA contribute to the accumulation of disability in SPMS as the disease evolves.^7^ Better tools and more research are needed to quantify inflammatory disease activity, to help clinicians better identify and discriminate between inflammatory disease activity and the progressive worsening that may currently be overlooked in patients considered to have non-active disease. Standardized and more accurate characterization of active and non-active cohorts using biomarkers,^26-32^ cognitive measures,^33^ electronic health technologies^34-37^ and standardized MRI testing^38^ may help.

Limitations of the Adelphi DSP^20^ and the EXPAND trial^21^ have been published previously; however, it is important to highlight that, in the current analysis, the definitions of disease activity differed between the two trials. First, assessments of the occurrence of relapse encompassed different timeframes. In Adelphi, the occurrence of relapse was assessed at index date over the previous year; for the EXPAND-PBO cohort, it was assessed at baseline over the previous 2 years and on study through recording relapse during follow-up. Secondly, the two trials used slightly different criteria for evidence of inflammatory activity on MRI. In Adelphi, patients were classified as ‘active’ if ≥1 Gd+ or a new T1 or T2 lesion was present in the most recent MRI scan. For the EXPAND-PBO cohort, patients were classified as ‘active’ at baseline if ≥1 Gd+ T1 lesion was present in the baseline MRI scan, while the presence of disease activity ‘on study’ was identified by the presence of Gd+ T1 and/or new/enlarging T2 lesions. Consequently, although no Gd+ lesions were present at baseline in the EXPAND-PBO non-active group, it is possible that T2 lesions were present; plausibly, the longer timeframe for pre-study relapse assessment in EXPAND-PBO may partially account for this potential difference in lesions at baseline compared with the Adelphi cohort at index date.

## Conclusion

In conclusion, our data highlight the difficulties in defining active versus non-active SPMS reliably and consistently. Our findings suggest the potential negative implications of less frequent monitoring of non-active disease, which could result in suboptimal management. MRI could be a better option than relapse for the identification of disease activity, however, our data suggest a monitoring gap that might affect the management of SPMS. Patients with non-active SPMS should be monitored more closely using MRI to ensure optimal disease detection and management.

## Data Availability

Adelphi Real-World DSP in MS analysis: The full dataset from which results reported in this manuscript are drawn remains proprietary to Adelphi Real-World and is not publicly available. Access to the raw dataset reported here may be granted on reasonable request to the corresponding author, dependent on the intended use and subject to third-party agreements. EXPAND-PBO analysis: The full dataset from which results reported in this manuscript are drawn is available from the corresponding author, upon reasonable request.
